# Detecting non-adjacent dependencies is the exception rather than the rule

**DOI:** 10.1371/journal.pone.0270580

**Published:** 2022-07-14

**Authors:** Laure Tosatto, Guillem Bonafos, Jean-Baptiste Melmi, Arnaud Rey

**Affiliations:** 1 CNRS, LPC, Aix Marseille Univ, Marseille, France; 2 ILCB, Aix Marseille Univ, Aix-en-Provence, France; 3 CNRS, Centrale Marseille, I2M, Aix Marseille Univ, Marseille, France; Abertay University, UNITED KINGDOM

## Abstract

Statistical learning refers to our sensitivity to the distributional properties of our environment. Humans have been shown to readily detect the dependency relationship of events that occur adjacently in a stream of stimuli but processing non-adjacent dependencies (NADs) appears more challenging. In the present study, we tested the ability of human participants to detect NADs in a new Hebb-naming task that has been proposed recently to study regularity detection in a noisy environment. In three experiments, we found that most participants did not manage to extract NADs. These results suggest that the ability to learn NADs in noise is the exception rather than the rule. They provide new information about the limits of statistical learning mechanisms.

## Introduction

Our environment is composed of continuous streams of stimuli, among which some correspond to invariant and repeated patterns. A central feature of our cognitive system is our sensitivity to the distributional properties of the environment inputs, allowing us to pick up on these patterns, also commonly referred to as *regularities*. The field of statistical learning (SL), sometimes also called *implicit* statistical learning [1], studies the mechanisms underlying the detection of regularities, generally unintentionally, and how this structures our perception and our understanding of the world [2–4]. As it is mostly an implicit or unintentional process, SL is usually viewed as an automatic phenomenon, in which individuals grasp knowledge of the input’s structure by mere exposure and use this knowledge to predict future events, e.g., [5], [6]. However, there is cumulative evidence showing that the context surrounding the regularity—its structure or characteristics—can hinder or facilitate SL so that exposure is not a sufficient condition.

Co-occurring regular events A and B can either appear systematically one after the other in the environment (adjacent dependency) or separated by random or unrelated events (non-adjacent dependency; NAD). NADs are naturally present in the environment and especially relevant in verbal material, for example in English, in phrases like “is writing”, where the auxiliary and the inflectional morpheme co-occur at a distance while the verb stem can vary [7]. NAD learning has been shown on a variety of materials, from musical or visual stimuli [8], [9] to artificial languages [10]. However, despite their relevance in structuring the environment, NADs seem difficult to detect and participants display poorer SL performances on NADs, despite the pattern being usually very simple, e.g., [11, 12], see [13], for a review.

Several factors have been identified as critical for learning NADs. For instance, the random events surrounding the regularity must not be too stable and the noise must be unstructured enough, so that individuals can extract the stable relationship between the distant elements of the regularity without learning the random events with the regularity [14]. Elements of the regularity must also be similar enough to one another and dissimilar enough compared to the noise for NAD detection to occur [12, 15]. Critically, drawing the attention of the participants towards the regularity (i.e., making them perform a specific task on the elements of the regularity) affects SL so that only the regularities processed attentionally are detected, regardless of the adjacency (or not) of its elements [16, 17].

Most studies exploring the limits of NAD extraction have used standard SL offline tasks. In these offline tasks, participants are first exposed to a visual or auditory familiarization stream comprising a set of regularities, followed by an offline test phase (e.g., judgement of familiarity on sequences that were or were not presented during the familiarization phase [18–20], or reaction time measures to familiar and unfamiliar stimuli, [21, 22]). Alternative experimental paradigms have also been developed to assess learning throughout the task by using online measures, such as recording serial RTs in serial response time tasks [23–25], in target detection tasks [26, 27], or in a self-paced familiarization task [28]. These approaches are useful to understand the learning trajectories of individuals and the learning dynamics of regularity extraction. They are also considered as more ecological learning situations since the regularities can be mixed up with random or unrelated information and participants are usually simply instructed to perform a task that does not explicitly request any learning of the stimulus material.

Rey et al. [29] recently proposed a novel Hebb-naming task to study the online extraction of an adjacent regularity mixed in with noisy information. In this adaptation of the standard Hebb paradigm, participants were shown a series of single letters displayed one at a time in the center of a computer screen and were simply instructed to name each letter. In the classic version of this paradigm, human adults are presented with sequences of digits for immediate serial recall. A given sequence (the Hebb sequence) is repeated every third trial and interposed by random (filler) sequences. Here, a single triplet of letters repeatedly appeared between random sequences of other letters and participants were not informed about the presence of this repeated pattern. They found that naming response times decreased for the predictable letters in the triplet (i.e., the second and third letters in the pattern) and this was interpreted as an index of anticipation, and therefore learning of the triplet pattern. However, results showed also that detection of the regularity only occurred under specific conditions: when the noise surrounding the pattern was unstructured enough (i.e., when random letters were drawn from a large enough set, different from the letters composing the pattern) and when the regularity was salient enough (e.g., when the regular pattern was composed of vowels and the random letters were consonants). Data also indicated that learning was enhanced when there was less space between two repetitions of the regular pattern.

In the present study, we used the same Hebb-naming task to determine whether NAD detection is possible in a stream of random noise. We used a regular pattern of two stimuli where the first (A) and the second (B) stimulus in the pattern were systematically separated by a random letter (X). Between two repetitions of the AXB pattern, a variable number of filler random consonant letters was presented to the participants. If participants were able to extract the NAD embedded in noise, then anticipatory shorter naming onsets should be observed on the predictable letter of the pattern (i.e., the second letter, B).

Testing experimentally the human ability to extract NADs is crucial because it is still claimed by some authors that it is a core feature of our statistical learning toolbox, e.g., [30]. However, this claim is at odds with most studies on NADs that have been reviewed recently by Wilson et al. [13]. This review article clearly states that “learning nonadjacent dependencies appears to be more cognitively demanding than detecting dependencies between adjacent elements, and only occurs in certain circumstances”. The present set of experiment was therefore conducted to test if the extraction of NADs is a general property of our cognitive system or if it is rather the exception than the rule.

### Overview of the experiments

Our study comprised three experiments that closely followed the logic of Rey et al. [29]. Instead of having a repeated sequence of three letters ABC, participants were exposed to a repeated non-adjacent dependency composed of two items A and B that were separated by a random item X (i.e., AXB). As in Rey et al. [29], a variable number of filler items was inserted between two repetitions of this regularity. Experiment 1 tested the detection of a NAD relationship between a regular pair of vowels constantly separated by a single random consonant. Between two repetitions of this NAD, there were from 1 to 3 random consonants selected from a set of 17 consonants. In Experiment 2, to make the regularity more salient, the regular pair was made up of two digits (instead of vowels) and the filler items were still composed of consonant letters. The number of random consonants was also slightly changed and varied from two to three to control for a possible learning bias. Finally, we used the same material in Experiment 3 as in Experiment 2, but we increased the length of noise filler sequences up to 3 to 5 random consonants. S1 Appendix recapitulates the experimental manipulations for each experiment.

## Experiment 1

### Method

All procedures in the present experiments involving human participants were performed in accordance with the ethical standards of the institutional and/or national research committee and with the 1964 Declaration of Helsinki and its later amendments or comparable ethical standards. Informed consent was obtained from each of the participants included in the study.

#### Participants

Twenty-four adults (7 males, 17 females, *M*_*age*_ = 21.4 years), all right-handed, native speakers of French and students at Aix-Marseille University, participated in the study in exchange for a course credit. All reported normal or corrected-to-normal vision and none reported a history of attention problems or reading disabilities.

#### Materials and procedure

The general method for the experiment is similar to the one used by Rey et al. [29]. The experiment was run on a portable computer equipped with a serial response box and an audio-technical microphone. The microphone was held by the participant at approximately 3 cm from the mouth.

In this naming task, administered via E-Prime 2.0 [31], participants were presented individual letters in 70-point Arial white font in the center of a computer screen with a black background. Participants were instructed to read each letter aloud as fast as possible. Speech onset and accuracy (scored zero for naming errors but also for microphone failures) were recorded for each letter. A given letter stayed on the screen until the microphone was triggered at speech onset. The next letter then appeared after a fixed ISI of 800ms.

Participants first performed a microphone test, which also served as a short familiarization with the task. It consisted of 12 randomly selected naming trials composed of consonants and vowels. The naming experiment then consisted of 3 blocks of 100 trials. Participants could take a short break after every block. After each block of trials, participants received oral feedback that they were performing well but had to try to speed up (this feedback was given independently of their actual performance or speed). This instruction was provided in order to encourage participants to use any cue (such as predictive patterns) that would help them perform the task more rapidly. In total, the naming task lasted approximately 10–15 minutes.

The regular pattern was composed of two vowels (drawn from A, E, O) and the random consonant letters were drawn from the set of B, C, D, F, G, H, J, K, L, M, N, P, R, S, T, V, Z (17 consonants). The regular pattern was repeated 17.86 times per block on average (*SD* = .26, *Min* = 17 and *Max* = 19), leading to a mean number of 53.58 repetitions (*SD* = .78, *Min* = 52 and *Max* = 55) per participant. The order of appearance of the consonants was random, whereas the two vowels composing the non-adjacent regularity were always presented in the same order and were separated by a single random consonant letter. For example, one participant could be assigned the following regularity A-*x*-O, where *x* represents a random consonant, and A and O would be the only two vowels he or she would encounter while performing the task. Each repetition of the regularity was always separated from the next one by 1 to 3 random consonants. The letters constituting the regularity and their serial order were counterbalanced across participants. For example, one participant could see the following sequence of letters, A-*x*-O being the repeated regularity: L-T-**A**-V-**O**-D-P-R-**A**-B-**O**-Z-**A**-C-**O**-N-G-**A**-S-**O**-…

For convenience purposes, hereafter, random consonants will be designated as “noise” and letters of the regularity will be referred to by their serial position in the regularity (i.e.: Position 1 for A and Position 2 for B in AXB). The only predictable event in the task was the appearance of the letter in Position 2 after Position 1 and a random consonant. Thus, in case of learning of the dependency, we expect a decrease in speech onset latencies (SOLs) specifically for Position 2 over several repetitions of the regularity (i.e., Position 1 > Position 2).

After performing the task, all participants responded to a short questionnaire. The experimenter asked them (1) “*Did you notice something particular in this experiment*? *YES/NO*” and, in case of a “*YES*” answer the follow-up question (1b) “*Can you explain what you noticed*?”. If a participant reported to have noticed the repeated presentation of a sequence or sequences of letters he or she was asked (1c) “*Can you recall which sequence(s) of letters was repeated*?”. In case the answer to the first question was “NO” or participants provided an explanation unrelated to the presence of repeated patterns, the experimenter explicitly asked the participant (2) “*Did you notice a repetition of a sequence of letters*? *YES/NO”* and “*Can you recall which sequence of letters was repeated*?”.

### Results

#### Questionnaire answers

Out of 24 participants, only one participant was fully aware of the presence of a regularity and recalled correctly the two letters of the regularity, mentioning that they were separated by a random letter. 10 participants reported that they did not notice anything particular while performing the task and 13 participants reported that they felt some letters were appearing more regularly but failed to report the NAD structure or the letters composing the NAD.

#### Speech onset latencies

**Fig 1:**
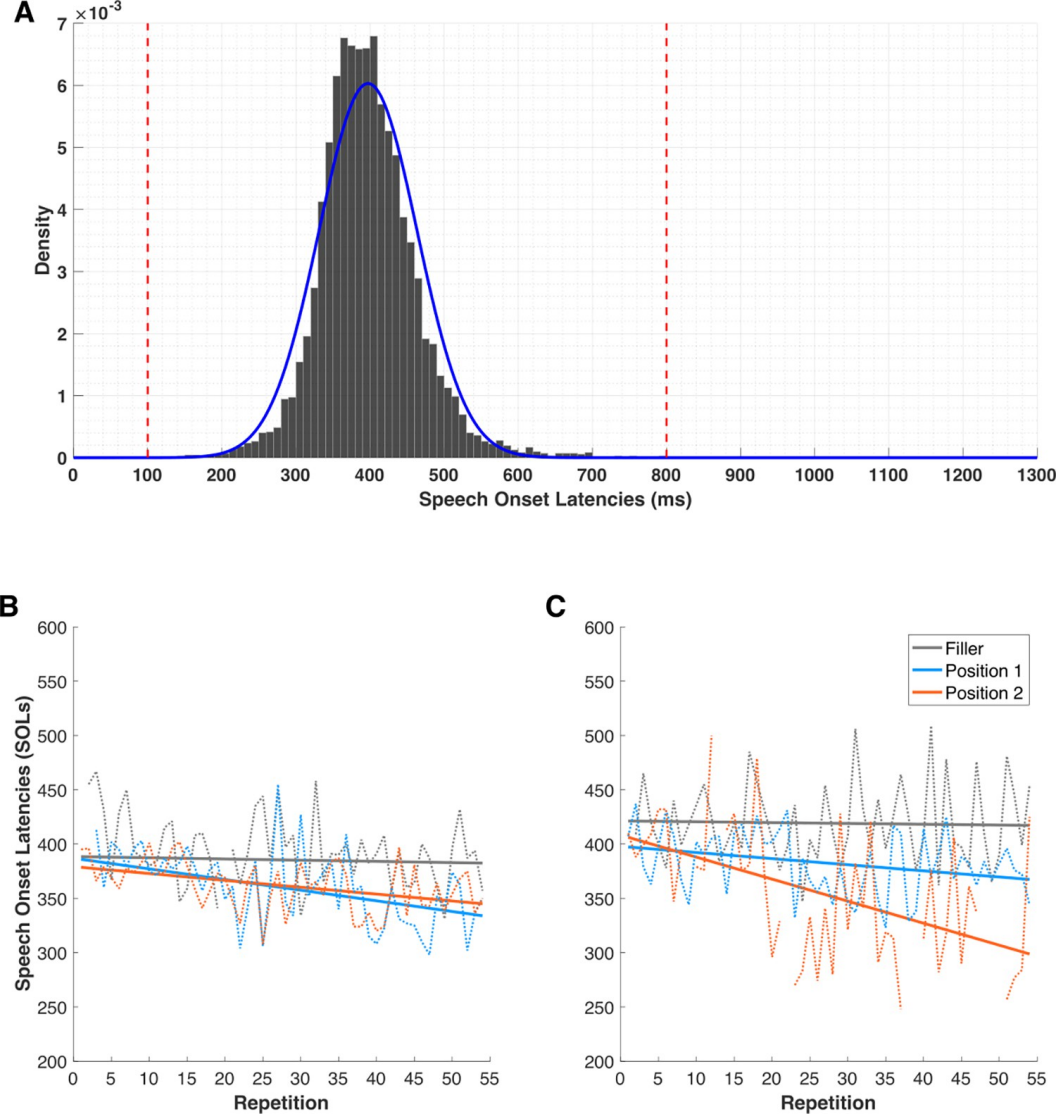
Speech onset latencies (SOLs) for Experiment 1. *Note*. Density plot of speech onset latencies (SOLs) showing the fitted normal distribution in blue, with a red line indicating our cut-off scores (Panel A). Speech onset latency (SOL) per position for each repetition (dotted lines) and fitted linear regressions (solid lines) in Participant 13 (Panel B) and Participant 11 (Panel C) who was the only participant to explicitly recall the NAD. Discontinuities correspond to missing values.

Only trials with correct naming responses were analyzed (96.4% of the data). Fig 1A shows that the distribution of speech onset latencies (SOLs) over all participants and trials was close to normal in our task. Visual inspection of the response times distribution suggested that responses faster than 100 ms or exceeding 800 ms were clearly outside the distribution leading to the exclusion of these responses (0.3% of the data). Additionally, we excluded for each participant data points deviating from the mean by plus or minus 2.5 times the standard deviation (2.4% of the data).

Bayes factors (BF) were used to assess the strength of evidence for the alternative hypothesis (learning of NAD), H1, over the null (absence of learning), H0 [32]. Conventionally, a BF greater than 3 will be considered as evidence for H1 rather than H0, and a BF smaller than 1/3 will be considered evidence for H0 rather than H1 [33, 34]. A BF-value between those two thresholds will be considered as anecdotal evidence [35].

To capture the evolution of SOLs across repetitions of the regularity, we computed the slope of the learning curve for each condition (noise letters, Position 1 and Position 2) for each participant. This was achieved by fitting a simple linear regression to the evolution of SOLs across repetitions, for each condition and each participant (Fig 1B is an example of fitting a simple linear regression to each condition for one participant). This dependent variable (i.e., learning slope) allowed us to study the evolution of RTs throughout the task (see S2 Appendix for the learning slopes obtained for each condition and for each participant in Experiment 1).

**Fig 2:**
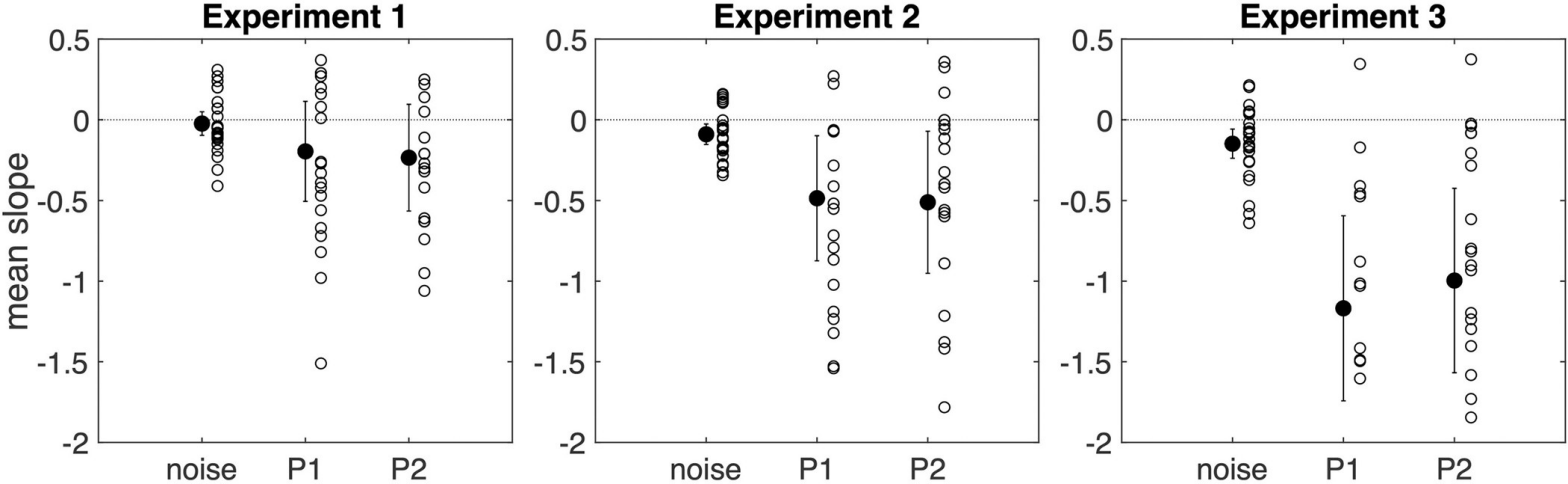
Mean slopes for the three conditions (Noise, Position 1, Position 2) in Experiment 1 to 3. *Note*. Mean (filled circle) and individual learning slope per Condition (Noise, Position 1, Position 2) for Experiment 1, Experiment 2 and Experiment 3. Error bars represent 95% confidence intervals.

The mean slopes for the three Conditions (Noise, Position 1, and Position 2) were respectively -0.02 (CI: 0.07), -0.20 (CI: 0.31) and -0.23 (CI: 0.33). A one-way repeated measures Bayesian ANOVA was conducted on the slopes with Condition as a within-participants factor. This analysis, with *BF*_*M*_ = 0.343, indicates anecdotal evidence for the null. Post-hoc comparisons further indicate that we have substantial evidence for the absence of difference between Position 1 and Position 2 (*BF*_*10*_ = 0.225, see Fig 2).

We also found that the participant who reported the regularity explicitly exhibits a decrease in SOLs for Position 2 that follows the expected response pattern (see Fig 1C). A repeated measures Bayesian ANOVA on the SOLs of this participant with Block (1, 2 or 3) and Position (1, or 2) as within-participant factors provides evidence of the learning of the NAD, as the model Block + Position + Block * Position is more likely than any other model (*BF*_*M*_ = 4.963) and a post-hoc comparison indicates a strong evidence for Position 1 > Position 2 (*BF*_*10*_ = 12.16).

### Discussion

The main result of Experiment 1 is an absence of significant acceleration of response times (i.e., SOLs) on the only predictable letter in the stream, i.e., the vowel situated in Position 2 of the regular pattern. Only one participant noticed the presence of this pattern and produced the expected result on response times. Almost all participants did not detect the non-adjacent dependency between the two vowels of the regular pattern.

This result is surprising considering that we thought these specific conditions were ideal for learning, in agreement with previous work. Indeed, Rey et al. [29] found that regularity extraction occurs more easily when occurrences of the regularity are separated by a small number of random letters and when the regularity is highly salient compared to the noise (e.g., when the regularity is composed of vowels and the noise is composed of consonants). Furthermore, in Rey et al. [29], the regularity was composed of three successive letters always appearing in the same order and only in the regularity, so that the last two letters were predictable, and participants had to learn two dependencies. In our experiment, there was only one dependency to learn, which should have been easier to detect.

In this first experiment, the learning slope for Position 1 and 2 was also not significantly different from the learning slope for the Noise condition. This result clearly indicates that most participants did not notice, nor implicitly extract, any difference between the occurrences of the vowels and the background noise consonants.

However, we also identified a potential bias in our experiment that could account for the absence of difference between Position 1 and 2. Indeed, because the number of random letters between two repetitions of the regularity could be drawn from 1 to 3, when only one letter was interposed, participants were exposed to the following sequence: A-*x*-O-*x*-A-*x*-O. In this specific case, A was followed by O, but O was also closely followed by A. The directionality of the dependency could be lost in these conditions and A could not be more predictive of O than the reverse.

Experiment 2 was conducted to cancel this bias by increasing the minimal distance between Position 2 and Position 1. Instead of interposing from 1 to 3 random consonant letters, we excluded the possibility of one consonant letter and restricted the number of interposed consonant letters from 2 to 3. We also changed a second feature in order to increase the saliency of the regularity: instead of vowels, we used a pair of digits surrounded by random consonant letters.

## Experiment 2

### Method

#### Participants

A different sample of twenty-four native French adults (5 males, 19 females, *M*_*age*_ = 21,6 years) participated in the experiment for course credit. Participants were all students at Aix-Marseille University, right-handed, had normal or corrected-to-normal vision, and reported no history of attention problems or reading disabilities.

#### Materials and procedure

The procedure was identical to Experiment 1. The random letters were also drawn from the same set of 17 consonants. However, the stimuli used to form the regularity were pairs of digits drawn from the following set of four digits: 2, 3, 5 and 7. The pairs constituting the regularity and their serial order were counterbalanced across participants. Another difference is the number of random letters inserted between repetitions of the regularity, varying from 2 to 3 consonants. The regular pattern was repeated 17.85 times per block on average (*SD* = .26, *Max* = 17 and *Min* = 19), leading to a mean number of 53.54 repetitions (*SD* = .78, *Max* = 52 and *Min* = 55) per participant.

### Results

#### Questionnaire answers

Out of 24 participants, three participants were fully aware of the presence of a regularity and correctly recalled the two digits of the regularity in the correct order, one of them even mentioning that repetitions of the pair were separated by 2 or 3 random letters. All other participants reported that they felt some characters were appearing more regularly but failed to recall the actual regularity.

#### Speech onset latencies

As in Experiment 1, only trials with correct naming responses were analyzed (97.9% of the data). Data points below 100 ms and over 800 ms (0.5% of the data) were excluded, as well as outliers deviating from the participant’s mean by over and under 2.5 times their standard deviation (2.3% of the data).

Again, we computed a linear regression on the SOLs for each Condition and each participant and used the slope of the regression as the dependent variable (see S3 Appendix). The mean slopes for the three Conditions (Noise, Position 1, and Position 2) were respectively -0.09 (CI: 0.06), -0.48 (CI: 0.39) and -0.51 (CI: 0.44). A one-way repeated measures Bayesian ANOVA was conducted on these slopes with Condition as a within-participant factor. This analysis did not provide evidence to speak of (*BF*_*M*_ = 0.634). However, post-hoc comparisons indicate substantial evidence for H_0_ and the absence of difference between Position 1 and Position 2 (*BF*_*10*_ = 0.218, see Fig 2).

As for the three participants who reported the regularity explicitly, we conducted repeated measures Bayesian ANOVAs on the SOLs of each participant with Block (1, 2 or 3) and Position (1, or 2) as within-participant factors. We found evidence for the learning of the NAD for only one participant for whom the model Block + Position is more likely than any other model (*BF*_*M*_ = 3.789) and a post-hoc comparison indicates a strong evidence for Position 1 > Position 2 (*BF*_*10*_ = 350.998).

### Discussion

Although we controlled for the distance between two repetitions of the regularity and we increased the saliency of the regular pair, we found—as in Experiment 1—no evidence of learning and no evidence that Position 2 could be predicted by Position 1. Three participants were able to explicitly extract the regularity but among them, only one presented the expected pattern on response times. The present results therefore suggest that extracting NADs in noise is possible but very demanding and most of the time, impossible.

In Experiment 3, we tried a final manipulation in order to increase the saliency of the regularity by increasing the distance between two repetitions of the NAD. Instead of having between 2 or 3 random letters between two repetitions of the NAD, we inserted from 3 to 5 random letters.

## Experiment 3

### Method

#### Participants

Twenty-four native French adults (one male, 23 females, *M*_*age*_ = 19,6 years) participated in this experiment for course credit. Participants were all students at Aix-Marseille University, right-handed, had normal or corrected-to-normal vision, and reported no history of attention problems or reading disabilities.

#### Materials and procedure

The procedure was identical to Experiments 1 and 2. The same stimulus material was used as in Experiment 2. The only difference was the number of random consonant letters interposed between repetitions of the regularity that varied randomly from 3 to 5 consonants. The regular pattern was repeated 14 times per block on average (*SD* = .28, *Min* = 13 and *Max* = 15), leading to a mean number of 42 repetitions (*SD* = .78, *Min* = 41 and *Max* = 44) per participant.

### Results

#### Questionnaire answers

Out of 24 participants, two participants recalled the regularity correctly. All other participants reported that they did not notice anything while performing the task.

#### Speech onset latencies

Only trials with correct naming responses were analyzed (97.1% of the data). Data points below 100ms and over 800ms (0.3% of the data) were excluded, as well as outliers deviating from the participant’s mean by over and under 2.5 times their standard deviation (2.4% of the data).

The mean slopes for the three Conditions (Noise, Position 1, and Position 2) were respectively -0.15 (CI: 0.09), -1.16 (CI: 0.57) and -1 (CI: 0.57). A one-way repeated measures Bayesian ANOVA was conducted on the slopes of these regressions with Condition as a within-participant factor. This analysis provides very strong evidence for H_1_, with *BF*_*M*_ = 56.71. Post-hoc comparisons indicate strong evidence for H1 when comparing Noise and Position 1, and Noise and Position 2 (respectively, *BF*_*10*_ = 50.68 and *BF*_*10*_ = 11.77) but provide substantial evidence for H_0_ and the absence of difference between Position 1 and Position 2 (*BF*_*10*_ = 0.258, see Fig 2 and S4 Appendix).

As for the two participants who reported the regularity explicitly, only one participant exhibits the expected response pattern, providing evidence of learning the NAD, as the model Block + Position + Block*Position is more likely than any other model (*BF*_*M*_ = 5.389) and a post-hoc comparison indicates a strong evidence for Position 1 > Position 2 (*BF*_*10*_ = 8180.531).

### Discussion

In Experiment 3, we increased the amount of noise between two repetitions of the regularity and this final manipulation did not produce any increase in learning of the NAD. Consistent with Experiment 1 and 2, only a minority of participants was able to detect the NAD.

## General discussion

In the present study, participants had to name aloud single letters and digits that were displayed one at a time on a computer screen as their SOLs were recorded. Unbeknownst to them, the stream contained a regular pair of stimuli that always occurred in the same order, separated by a single random stimulus. In three experiments, we did not find any evidence indicating that participants had learnt the regularity and could predict the second element on the basis of the first. In each experiment, only a very small number of participants explicitly detected the regularity and displayed greater negative slopes on SOLs for Position 2 relative to Position 1.

Although we identified a potential bias in Experiment 1 due to the occurrence of cases in which two repetitions of the regularity was separated by only one random consonant letter, this bias was corrected in Experiment 2 and even more in Experiment 3. Our effort to increase the saliency of the regularity in Experiment 2 by replacing vowels by digits did not produce any evidence of NAD learning. Most participants were unable to detect NADs and could not predict the appearance of the second stimulus based on the first one.

This new set of experiments joins a long series of other experiments showing that extracting NADs is very challenging for our cognitive system and depends on factors others than the one tested here. For example, contrary to Pacton and Perruchet (2008) who showed that a critical factor for NADs to be learnt is to force the simultaneous attentional processing of the two elements composing the NADs, we did not use here this manipulation to help participants extract the regularity. Conversely, we found that when there is no specific task to perform on the regularity and when the NAD is embedded within a stream of continuous information, participants have trouble detecting this regularity.

The present results are therefore consistent with several studies stating that mere repetition of a NAD pattern is not a sufficient condition to statistical learning. A high signal to noise ratio does not seem to suffice either because in our experiments, the NAD was made very salient compared to the random noise. In our case, even if our experimental parameters were designed to make the regularity “stand out”, it did not suffice to help participants detect the non-adjacent dependency.

The critical role of attentional processing in SL is not a new idea, e.g., [9, 16, 36], and our study provides further evidence that, in ecological conditions were patterns are embedded in noise and dependencies are non-adjacent, if attentional resources are not particularly directed towards the relevant information, it is extremely difficult to extract NADs. It therefore suggests that extracting NADs is the exception rather than the rule.

The present data therefore join a recent review article about NADs [13] that clearly stipulates that the extraction of NADs cannot be considered as a general cognitive ability that is ready to go and that we use frequently and effortlessly. Computation models that assume NADs extraction as a core feature of their theoretical proposition are certainly overestimating our ability to memorize and encode long-distance dependencies, e.g., [30]. This cautious claim does not mean that extracting NADs is impossible. It is possible but it is rare and it occurs only under very specific conditions.

One can also note from Experiment 1 to 3 that learning slopes for Position 1 and 2 tended to increase. While there was no difference with the noise condition in Experiment 1, a clear difference between Noise and Position 1 or 2 appeared in Experiment 3. These greater negative slopes for Positions 1 and 2 suggest that participants responded faster to these items along the course of the experiment. Because no difference was observed between Position 1 and 2, this increase in learning slope is likely due to a frequency effect, the two digits of the NAD being not only more salient but also more frequent compared to the consonant letters. Participants therefore probably developed an expectation for the two digits which produced this equal facilitation on SOLs for Position 1 and 2. The fact that the digits of the NAD ended up standing out from the noise consonants did not however allow participants to grasp the non-adjacent dependency.

It is certainly useful to mention here that the absence of NAD extraction in the present set of experiments is also likely due to some specific features of the Hebb-naming paradigm that we used. Rey et al. [29] indeed reported that even adjacent regularities were not obvious to extract in this task. From the experimenter’s point of view, the presence of a regularity is noticeable but from the participant’s perspective, it seems to be less evident. Indeed, their task is mainly to read aloud the single letters that appear successively on the screen. Encoding the repetition of a serial pattern of items requires to pay attention to this pattern, which is not required here to perform the task and may be irrelevant to the participant. This surely limits the ability of participants to notice and extract these regularities.

However, a few participants managed to explicitly report the NAD indicating that they were able to go beyond the processing of each single letter. Among these participants, not all produced the expected response time pattern (i.e., faster RTs on the predictable position, the B for an AXB pattern). Although the dissociation between explicit and implicit learning is frequently reported in the field of statistical learning, it is usually reversed: implicit learning is observed while explicit learning is absent. In the present situation, having explicitly noticed the occurrence of a NAD pattern may not be always beneficial to the processing of the predictive item. This is likely due to the random letter that always separates the A and B elements. Due to this variability, it may repeatedly create some uncertainty or surprise that prevent the participant from using the explicit knowledge that A is apparently always followed by B and prevents speeding up when B appears.

These issues raise the larger question of the role of individual differences in SL. Some studies suggest that the heterogeneity of SL performances may be related to differences in the cognitive abilities of participants. For instance [37], found strong intercorrelations between statistical learning (both in adjacent and non-adjacent regularity learning), verbal working memory, and language comprehension. No direct link has yet been established between more general abilities, such as intelligence, and performances during SL tasks, as [38] found no link between IQ-related tasks scores (e.g., Raven’s Advanced Progressive Matrices; digit span; a verbal working memory task) and a variety of SL tasks (visual/auditory, verbal/non-verbal, adjacent/non-adjacent regularities tasks). However, this absence might be connected to a ceiling effect in participant’s performances in SL. Therefore, an interesting perspective in the field of SL would be to study the relationship between IQ and more complex and ecological SL tasks, such as the one presented in the present paper. It may help us understand the cognitive characteristics of the few participants who manage to detect non-adjacent dependencies.

To conclude, the present study shows the limits encountered by the cognitive system to extract NAD patterns in a continuous stream of noisy stimuli. Out of three studies and despite various experimental manipulations made on the saliency of the pattern, only 6 participants out of 64 were able to extract the NAD pattern. Additionally, only 3 of these participants used this knowledge to predict the end of the pattern given its beginning. Our results clearly show that NAD learning is not automatic and requires both attention and relevance to the task in progress to be processed. They challenge the traditional view that NADs are easily extracted in a stream, especially a speech stream, and later used by the individual to adapt to the environment or produce correct utterances. Overall, this study supports the idea that the extraction of NADs is a not general property of our cognitive system.

## Supporting information

S1 Appendix Overview of the experiments. (DOCX)

S2 Appendix Learning slopes per condition (noise, Position 1 and 2) and for all participants in Experiment 1 (calculated from linear regressions). (DOCX)

S3 Appendix Learning slopes per condition (noise, Position 1 and 2) and for all participants in Experiment 2 (calculated from linear regressions). (DOCX)

S4 Appendix Learning slopes per condition (noise, Position 1 and 2) and for all participants in Experiment 3 (calculated from linear regressions). (DOCX)
