# Supplementary material for: Detecting non-adjacent dependencies is the exception rather than the rule
Source: PLoS One. 2022 Jul 14;17(7):e0270580. doi: 10.1371/journal.pone.0270580 (PMC9282578; doi:10.1371/journal.pone.0270580)
Supplement: S1 Appendix — (DOCX) [file pone.0270580.s001.docx]

**Appendix A**

*Overview of the experiments*

|  |  | Regularity | Random noise | Number of random letters  between repetitions |
| --- | --- | --- | --- | --- |
| Experiment 1 |  | Vowels | Consonants | 1 to 3 |
| Experiment 2 |  | Digits | Consonants | 2 to 3 |
| Experiment 3 |  | Digits | Consonants | 3 to 5 |
