# Supplementary material for: Detecting non-adjacent dependencies is the exception rather than the rule
Source: PLoS One. 2022 Jul 14;17(7):e0270580. doi: 10.1371/journal.pone.0270580 (PMC9282578; doi:10.1371/journal.pone.0270580)
Supplement: S2 Appendix — (DOCX) [file pone.0270580.s002.docx]

**Appendix B**

*Learning slopes per Condition (noise, Position 1 and 2) and for all participants in Experiment 1 (calculated from linear regressions)*

| Participant | Noise | Position 1 | Position 2 |
| --- | --- | --- | --- |
| 1 | 0.2 | 1.02 | 0.77 |
| 2 | 0.07 | 0.27 | 0.25 |
| 3 | -0.04 | -0.33 | -0.42 |
| 4 | 0.02 | 0.01 | -0.27 |
| 5 | -0.23 | -0.67 | -0.63 |
| 6 | -0.31 | -1.51 | -0.95 |
| 7 | 0.27 | 0.37 | 1 |
| 8 | 0.07 | -0.72 | -0.32 |
| 9 | -0.05 | -0.27 | -0.11 |
| 10 | 0.11 | 0.08 | 0.97 |
| 11 | -0.08 | -0.56 | -2.03 |
| 12 | -0.09 | 0.16 | -0.74 |
| 13 | -0.11 | -0.98 | -0.63 |
| 14 | 0.2 | 0.29 | -0.3 |
| 15 | -0.08 | -0.42 | 0.14 |
| 16 | -0.15 | -0.47 | -0.21 |
| 17 | -0.41 | -2.12 | -2.16 |
| 18 | -0.12 | 0.2 | 0.69 |
| 19 | 0.31 | 1.52 | 0.22 |
| 20 | -0.1 | -0.39 | -0.61 |
| 21 | 0.24 | 0.29 | 0.91 |
| 22 | -0.19 | -0.82 | -1.06 |
| 23 | -0.05 | 0.64 | -0.21 |
| 24 | -0.04 | -0.26 | 0.05 |
| Mean (CI) | -0.02 (0.07) | -0.20 (0.31) | -0.23 (0.33) |
