# Supplementary material for: Detecting non-adjacent dependencies is the exception rather than the rule
Source: PLoS One. 2022 Jul 14;17(7):e0270580. doi: 10.1371/journal.pone.0270580 (PMC9282578; doi:10.1371/journal.pone.0270580)
Supplement: S3 Appendix — (DOCX) [file pone.0270580.s003.docx]

**Appendix C**

*Learning slopes per Condition (noise, Position 1 and 2) and for all participants in Experiment 2 (calculated from linear regressions)*

| Participant | Noise | Position 1 | Position 2 |
| --- | --- | --- | --- |
| 1 | -0.17 | -0.52 | 0 |
| 2 | -0.18 | -0.87 | -1.38 |
| 3 | 0 | 0.9 | 0.17 |
| 4 | -0.18 | -2.3 | -3.73 |
| 5 | 0.11 | -1.02 | -1.22 |
| 6 | 0.15 | 0.6 | 1.26 |
| 7 | -0.12 | -1.19 | -1.42 |
| 8 | 0.14 | 1.18 | 1.21 |
| 9 | -0.22 | -1.54 | -0.42 |
| 10 | -0.04 | -0.28 | -0.06 |
| 11 | -0.07 | -0.07 | 0.52 |
| 12 | -0.27 | -0.41 | -0.89 |
| 13 | -0.11 | -0.06 | -0.03 |
| 14 | 0.11 | 1.01 | -0.18 |
| 15 | -0.17 | -1.32 | -0.58 |
| 16 | -0.34 | -1.53 | -2.42 |
| 17 | -0.32 | -1.23 | -0.56 |
| 18 | -0.17 | -0.79 | -0.6 |
| 19 | -0.06 | 0.72 | -0.32 |
| 20 | 0.16 | 0.27 | 0.33 |
| 21 | -0.05 | 0.22 | -0.11 |
| 22 | 0.13 | -0.72 | 0.36 |
| 23 | -0.28 | -0.55 | -0.4 |
| 24 | -0.19 | -2.15 | -1.78 |
| Mean (CI) | -0.09 (0.06) | -0.49 (0.39) | -0.51 (0.44) |
