# Supplementary material for: Detecting non-adjacent dependencies is the exception rather than the rule
Source: PLoS One. 2022 Jul 14;17(7):e0270580. doi: 10.1371/journal.pone.0270580 (PMC9282578; doi:10.1371/journal.pone.0270580)
Supplement: S4 Appendix — (DOCX) [file pone.0270580.s004.docx]

**Appendix D**

*Learning slopes per Condition (noise, Position 1 and 2) and for all participants in Experiment 3 (calculated from linear regressions)*

| Participant | Noise | Position 1 | Position 2 |
| --- | --- | --- | --- |
| 1 | -0.07 | -0.46 | -0.62 |
| 2 | -0.64 | -2.17 | -1.85 |
| 3 | -0.35 | -3.3 | -1.4 |
| 4 | 0.21 | 1.14 | 1.67 |
| 5 | -0.15 | -1.6 | -0.82 |
| 6 | 0.09 | -0.47 | -0.21 |
| 7 | 0.05 | -0.17 | -0.28 |
| 8 | -0.58 | -3.58 | -3.13 |
| 9 | -0.02 | -2.47 | 0.72 |
| 10 | -0.16 | -1.42 | -0.9 |
| 11 | -0.09 | 0.6 | 0.37 |
| 12 | -0.12 | -0.88 | -0.02 |
| 13 | -0.19 | -2.39 | -1.3 |
| 14 | 0.05 | 0.35 | -0.8 |
| 15 | -0.05 | -1.01 | -0.08 |
| 16 | 0.2 | 0.81 | -1.58 |
| 17 | -0.26 | -1.5 | -1.2 |
| 18 | -0.26 | -1.49 | -0.04 |
| 19 | -0.53 | -2.3 | -5.37 |
| 20 | 0.04 | 1.41 | -0.08 |
| 21 | -0.17 | -0.41 | -1.24 |
| 22 | -0.08 | -1.03 | -0.93 |
| 23 | -0.08 | -2 | -1.73 |
| 24 | -0.37 | -3.69 | -3.11 |
| Mean (CI) | -0.15 (0.09) | -1.17 (0.57) | -0.99 (0.57) |
